# Oxidative Stress and Its Consequences in the Blood of Rats Irradiated with UV: Protective Effect of Cannabidiol

**DOI:** 10.3390/antiox10060821

**Published:** 2021-05-21

**Authors:** Michał Biernacki, Małgorzata Michalina Brzóska, Agnieszka Markowska, Małgorzata Gałażyn-Sidorczuk, Bogdan Cylwik, Agnieszka Gęgotek, Elżbieta Skrzydlewska

**Affiliations:** 1 Department of Analytical Chemistry, Medical University of Bialystok, A. Mickiewicza 2D, 15-222 Bialystok, Poland; michal.biernacki@umb.edu.pl (M.B.); agnieszka.markowska@umb.edu.pl (A.M.); agnieszka.gegotek@umb.edu.pl (A.G.); 2 Department of Toxicology, Medical University of Bialystok, A. Mickiewicza 2C, 15-089 Bialystok, Poland; malgorzata.brzoska@umb.edu.pl (M.M.B.); malgorzata.galazyn-sidorczuk@umb.edu.pl (M.G.-S.); 3 Department of Pediatric Laboratory Diagnostics, Medical University of Bialystok, J. Waszyngtona 17, 15-269 Białystok, Poland; cylwikb@umb.edu.pl

**Keywords:** UV radiation, antioxidant therapy, cannabidiol, nude rats, in vivo model, blood plasma, polymorphonuclear leukocytes (PMNs), oxidative stress, lipid peroxidation, endocannabinoids

## Abstract

UVA/UVB radiation disturbs the redox balance of skin cells, and metabolic consequences can be transferred into the blood and internal tissues, especially after chronic skin exposure to UV radiation. Therefore, the aim of this study was to evaluate the effect of cannabidiol (CBD), an antioxidant and anti-inflammatory phytocannabinoid, on oxidative stress and its consequences in the blood of nude rats whose skin was exposed to UVA/UVB radiation for 4 weeks. It was shown that CBD penetrated the blood and in UVB-irradiated rats was preferentially located in the membranes of polymorphonuclear leukocytes, which promoted reduction of ROS generation and up-regulation of antioxidant ability by increasing the activity of glutathione reductase and thioredoxin reductase, while the level of reduced glutathione decreased by UV radiation. Consequently, reduction in UV-induced lipid peroxidation, assessed as 4-hydroxynonenal (4-HNE) and 8-isoprostane (8-isoPGF2α) as well as protein modifications, estimated as 4-HNE-protein adducts and protein carbonyl groups, was observed. CBD, by countering the UV-induced down-regulation of 2-arachidonylglycerol, promoted its antioxidant/anti-inflammatory effects by reducing CB1 and increasing PPARγ receptor activation and consequently ROS and TNF-α down-regulation. The results suggest that CBD applied topically to the skin minimizes redox changes not only at the skin level, but also at the systemic level.

## 1. Introduction

Ultraviolet radiation, a component of sunlight, is also the most common physical factor affecting the functioning of human skin [1]. The UV radiation reaching the earth’s surface consists of UVA and UVB rays, which differ in energy and consequently reach different layers of the skin. UVB radiation penetrates mainly into the epidermis, affecting the metabolism of its cells, including the basic cells of this layer, such as keratinocytes, while UVA radiation reaching the dermis modulates the metabolism of fibroblasts and dendritic cells, as well as other immune cells infiltrating the skin [2]. UVA radiation also reaches the micro vessels of the circulatory system; hence the secondary effects of this radiation can be transferred to the blood vessels, thus affecting the condition of the whole organism [3].

The most common effect of UVA and UVB radiation is the increased generation of reactive oxygen species (ROS)/reactive nitrogen species (RNS) in skin cells, and has been documented in vitro, ex vivo, and in vivo analysis [4,5,6]. These reactive species intensify the NF-κB-dependent pro-inflammatory signaling pathways, which increases the transcription of pro-inflammatory cytokines [7]. Moreover, by promoting the interaction of NF-κB and Nrf2 transcription factors, ROS indirectly modulate the transcriptional activity of Nrf2, resulting in a decrease in the biosynthesis of cytoprotective proteins including antioxidants dependent on the Nrf2/ARE pathway [8]. This promotes the formation of oxidative stress, which leads to the interaction of ROS with endogenous compounds, including macromolecules, such as DNA, proteins and lipids, causing oxidative modifications [9]. As a result, metabolically essential proteins lose their functional activity and also become less susceptible to degradation. This leads to the accumulation of non-functional proteins [10]. On the other hand, changes in the structure of lipids not only disturb the functions of cell membranes, but also affect cell signaling, in which participates products of lipid peroxidation and products of enzyme-dependent metabolism such as eicosanoids and endocannabinoids [4]. Thus, UV radiation is a causative factor, at the molecular level, of various skin lesions, including photoaging and the development of skin cancer, which accounts for about 40% of all cancers [11,12,13]. Two types predominate among skin cancers: non-melanoma skin cancers and melanoma, the most aggressive and lethal form [13,14], with an estimated 60–70% of cutaneous malignant melanomas caused by exposure to UV radiation [15]. This is all the more important due to the fact that UV radiation is also used in the phototherapy of skin diseases such as psoriasis [16].

Metabolic disorders resulting from UV radiation include not only local changes in the original site of damage (in skin cells), but through intercellular interactions their effects can be transferred to the bloodstream [3,17]. However, only a limited number of reports describe the effect of UV radiation on tissues other than the skin [3,17]. For this reason, it is important to identify the direction of metabolic changes, including redox balance and lipid metabolism, under the influence of UV radiation and to indicate a compound that can protect organism against these secondary effects of UV radiation.

Among the natural antioxidant/anti-inflammatory compounds that have been extensively researched recently are phytocannabinoids such as palmitoylethanolamide (PEA) found in egg yolk, soybean and peanut oil [18] as well as cannabidiol (CBD) found in *Cannabis sativa* L. [19]. Importantly, CBD is not a psychoactive compound, unlike most phytocannabinoids, which allows it to be used in higher doses without side effects [20].

To date, CBD has been shown to exhibit antioxidant properties when applied: in vitro to cultured skin cells; ex vivo to skin cells of psoriatic patients; and in vivo to UV-treated rat skin [5,21,22,23]. It was found that the application of CBD following exposure to UV (especially UVB) enhances the accumulation of this phytocannabinoid in skin cells, mainly in cell membranes [21]. CBD counteracts changes in redox balance by modifying the level/activity of both pro-oxidant and antioxidant compounds [5,21]. Moreover, by modifying the level of endocannabinoids, CBD indirectly regulates the activation of cannabinoid receptors involved in the regulation of ROS and TNF-α levels [19] and consequently, this phytocannabinoid reduces both redox imbalance and inflammatory processes. This effect is also the result of modulation of the interactions of transcription factors Nrf2 and NF-κB [24]. In consequence, by reducing oxidative conditions, CBD modifies lipid metabolism, especially in relation to skin cells exposed to UV radiation [24,25].

Pre-clinical studies to date indicate that topical application of CBD may be an effective treatment/supportive treatment for certain skin diseases such as eczema, psoriasis, pruritus and inflammation, but the underlying molecular mechanisms have not yet been fully identified [26,27]. However, it was found that the topical application of CBD-containing ointment to patients with psoriasis or atopic dermatitis improves skin hydration and elasticity, which non-invasively improves the quality of life of patients [27]. Topical application of CBD oil has also been shown to be an effective alternative to the treatment of peripheral neuropathy [28].

However, there is no data yet on the effects of CBD applied to the skin on metabolic changes, including redox balance in the blood of animals or humans. Therefore, the aim of this study was to evaluate the metabolic disturbances in the blood of nude rats chronically irradiated with UVA or UVB rays, as well as the potential protective effect of topical CBD application on metabolic processes affected by radiation. Due to the spread of CBD action over time and to maintain constant cell access to this compound, CBD was applied every 12 h. The results of these studies allowed the demonstration of cannabidiol in the blood of animals and the evaluation of redox balance changes both directly (by measuring ROS levels and antioxidants level/activity) and indirectly (by estimating the effects of oxidative stress on lipid metabolism) based on the level of lipid peroxidation products affecting on the proteins and endocannabinoids through receptors regulating the level of ROS and TNFα.

## 2. Materials and Methods

### 2.1. Animal Experiment

The experiment was carried out on male nude rats (Hsd:RH-Foxn1rnu) aged 8–9 weeks and weighing 260–302 g (Vivari s.c., Warsaw, Poland). Rats were housed under standard conditions on 12 h light/12 h dark cycles, and fed pellets containing a mixture of nutrients such as proteins, fiber and minerals [29]. All animal procedures were approved by the Local Ethics Committee for Animal Experiments in Olsztyn, Poland (Resolution No. 37/2019 of 26 April 2019).

The rats were divided into the following groups and skin of the rat’s back was exposed to the physical and chemical treatments, for a period of 4 weeks:

[control] group: non-toxic hydrophilic petrolatum, applied topically for 20 min every 12 h (*n* = 6);

[CBD] group: CBD (THC Pharm GmbH, Frankfurt, Germany) (120 mg/kg of body weight (b.w.); 2.5% *w*/*w* in petrolatum), [30], applied topically for 20 min every 12 h (*n* = 6);

[UVA] group: UVA radiation (365 nm) every 48 h (dose ncreasing from 0.5 to 5 J/cm^2^) (*n* = 6);

[UVA + CBD] group: UVA radiation (365 nm) every 48 h (dose increasing from 0.5 to 5 J/cm^2^ and CBD (120 mg/kg of b.w.; 2.5% *w*/*w* in petrolatum) applied topically for 20 min every 12 h (*n* = 6);

[UVB] group: UVB radiation (312 nm) every 48 h (dose increasing from 0.02 to 2 J/cm^2^) (*n* = 6);

[UVB + CBD] group: UVB radiation (312 nm) every 48 h (dose increasing from 0.02 to 2 J/cm^2^) and CBD (120 mg/kg of b.w.; 2.5% *w*/*w* in petrolatum) applied topically for 20 min every 12 h (*n* = 6).

The literature indicate that CBD was applied topically to the skin of animals in the dose range of 0.6 to 62.3 mg of CBD [30]. In this study, 31–36 mg of CBD was applied to the back of each rat, corresponding to 120 mg/kg body weight.

UV irradiation was carried out using a Cosmedico lamp (Stuttgart, Germany), designed for use in phototherapy of human skin diseases. Constant conditions were required to reproducibly deliver the specified dose of radiation and at the same time to protect the skin from overheating/burns. Plastic combs were therefore used to maintain a constant distance of approx. 2 cm between the lamp and the skin.

At the end of the experiment, animals were anesthetized by inhalation of isoflurane. Blood was collected into tubes with EDTA as an anti-coagulant and butylhydroxytoluene (BHT) as an antioxidant. Animals were sacrificed by cardiac excision.

Blood samples were centrifuged across a density gradient. In the first stage, blood was centrifuged at 3000× *g* at 4 °C for 20 min to obtain plasma and the buffy coat. In order to obtain polymorphonuclear leukocytes (PMNs), the buffy coat was further centrifuged using Gradisol G (Aqua-Med ZPAM–KOLASA, Łódz, Poland). Samples were layered on Gradisol G and centrifuged at 300× *g* for 25 min at room temperature. Cell fractions were collected, washed, and re-suspended in PBS containing a proteasome inhibitors mix. Cell purity was confirmed microscopically (Nikon Eclipse Ti, Nikon Instruments Inc., Melville, NY, USA). Isolated fractions of PMNs were lysed by sonication on ice, and stored at −80 °C for subsequent analysis.

All parameters examined in plasma are expressed per ml, while cellular parameters examined in PMNs are expressed per mg of protein (as determined according to the Bradford method [31]).

### 2.2. Methods

#### 2.2.1. Determination of Blood Plasma Basic Biochemical Parameters

In blood plasma basic biochemical parameters were determined using routine laboratory methods with commercial kits (Roche Diagnostics GmbH, Mannheim, Germany) on a COBAS c501 analyser (Roche/Hitachi, Tokyo, Japan) and the results are shown in Table 1. In detail, total protein was measured by colorimetric method, glucose was determined by enzymatic: glucose oxidase/peroxidase method, aspartate aminotransferase (AST) and alanine aminotransferase (ALT) were assayed by kinetic method with NADH and TRIS buffer without pyridoxal phosphate, triglycerides by enzymatic: lipoprotein lipase/glycerol kinase/glycerol-3-phosphate oxidase/peroxidase method, total cholesterol by enzymatic: cholesterol esterase/cholesterol oxidase/peroxidase colorimetric method, urea by enzymatic: urease/glutamate dehydrogenase method and electrolytes (Na^+^ and K^+^) by potentiometric method with ion-selective electrodes (ISE Indirect).

#### 2.2.2. Determination of CBD Level in Plasma and in the Cytosolic/Membrane Fraction of PMNs

CBD level was determined using liquid chromatography-tandem mass spectrometry LC-MS/MS (LC-MS 8060, Shimadzu, Kyoto, Japan) [32]. Compounds for analysis were obtained using the solid phase extraction (SPE) technique. CBD was separated on an Agilent Poroshell 120 EC-C18 analytical column (3.0 × 150 mm; 2.7 µm particle size). Flow rate was maintained at 0.8 mL/min, and 5 µL of sample was loaded onto the column. The autosampler was maintained at 4 °C, and the analytical column was maintained at 18 °C. The mobile phase consisted of 0.1% formic acid in water (eluent A) and 0.1% formic acid in acetonitrile (eluent B). Gradient steps were applied according to the following percentage changes and interval durations: 70% eluent B to 80% eluent B over 5 min; 80% B to 88% B over 10 min; 88% B to 100% B over 6 min; a hold was applied at 100% B for 4 min; 100% B to 70% B over 1 min; then a final hold was applied at 70% B to re-equilibrate the column for 4 min until the end of the run time. Electrospray ionization (ESI) was used in positive mode for multiple reaction monitoring (MRM) and quantification of each analyte. CBD-d_9_ (Cayman Chemical, Ann Arbor, Michigan, USA) was used as an internal quantification standard. The transitions of precursor to the generated ion were as follows: mass-to-charge ratio (*m*/*z*) 315.1 → 193.0 for CBD; and *m*/*z* 324.1 → 202.2 for CBD-d_9_. CBD levels are expressed in pmol/mL for plasma, and in pmol/mg protein for PMNs.

### 2.3. Redox Balance

#### 2.3.1. Determination of ROS Levels

Generation of ROS in either whole-blood or in PMNs was detected using an electron spin resonance (ESR) e-scan spectrometer (Noxygen GmbH/Bruker Biospin GmbH, Germany). The interaction of the spin probe CMH (1-hydroxy-3-methoxy-carbonyl-2,2,5,5-tetramethylpyrrolidine, 200 μM) with ROS was selected as an indicator, forming a stable nitroxide CM-radical (half-life: 4 h). Thus, ROS generation was determined on the basis of nitroxide accumulation kinetics in accordance with the electron spin resonance amplitude of the low-field component of ESR spectra [33]. ROS levels are expressed in nmol/mL for blood samples, and in nmol/min/mg protein for PMNs.

#### 2.3.2. Determination of the Activity of Antioxidant Enzymes

The activity of copper- and zinc-dependent superoxide dismutase (Cu, Zn-SOD–EC.1.15.1.1) ions was measured according to the method of Misra and Fridovich [34] as modified by Sykes [35]. One unit of enzyme activity was considered to be the amount of enzyme which inhibits epinephrine oxidation by 50%. Results are expressed in units of enzyme activity per ml of plasma.

The activity of glutathione peroxidase (GSH-Px–EC.1.11.1.6) was determined by spectrophotometric measurement at 340 nm of the conversion of NADPH to NADP^+^ per min at pH 7.4 [36]. One unit of GSH-Px activity was defined as the amount of enzyme catalyzing the oxidation of 1 mmol NADPH min^−1^ at 25 °C and pH 7.4. Results are expressed in units of enzyme activity per ml of plasma.

The activity of glutathione reductase (GSSG-R–EC.1.6.4.2) was determined by spectrophotometric measurement at 340 nm of the reduction of NADP^+^ to NADPH [37]. One unit of enzyme activity was specified as the amount that catalyzes the reduction of 1 µmol NADP^+^ per min at pH 7.4. Results are expressed in units of enzyme activity per ml of plasma.

The activity of thioredoxin reductase (TrxR–EC.1.8.1.9) was determined by spectrophotometric measurement using a commercial assay (Sigma-Aldrich, St. Louis, MO, USA) according to the included instructions [38]. One unit of enzyme activity was specified as the amount that catalyzing the oxidation of 1 µmol NADPH per min at 25 °C and pH 7.0. Results are presented in units of enzyme activity per ml of plasma.

#### 2.3.3. Determination of the Level of Non-Enzymatic Antioxidants

Glutathione (GSH) level was determined by capillary electrophoresis. Plasma components were separated on a capillary with an effective length of 40 cm operated at 27 kV with UV detection at 200 ± 10 nm [39]. GSH level was estimated using a calibration curve with a range of 1–120 nmol/mL (*r*^2^ = 0.9985).

Thioredoxin (Trx) levels were determined by ELISA (Enzyme Linked ImmunoSorbent Assay) [40]. Plasma samples were added to cover the bottom of a 96-well plate, and incubated overnight at 4 °C with primary antibody against thioredoxin (Abcam, Cambridge, MA, USA). Goat anti-rabbit was used as a labelled secondary antibody (Dako, Carpinteria, CA, USA). After washing, chromogen substrate was applied (3,3′,5,5′-tetramethyl-benzidine at 0.1 mg/mL) and absorption was measured at 450 nm. Trx level was estimated using a calibration curve with a range of 1–5 µmol/mL (*r*^2^ = 0.9979).

Plasma levels of ascorbic acid (vitamin C) were determined using the HPLC method described by Ivanovic [41]: plasma was mixed with an equivalent volume of metaphosphoric acid (100 g/L) and precipitated proteins were removed before analysis by centrifugation (1000× *g*; 10 min), then separated on an RP-18 column, and UV detection was conducted at 250 nm. The vitamin C levels were estimated using a calibration curve with a range of 2–50 µmol/mL (*r*^2^ = 0.9987) for vitamin C.

Vitamin A and E levels were determined according to the method of DeLeenher [42]. Lipophilic vitamins (A and E) were extracted from plasma using hexane containing 0.025% butylated hydroxytoluene. The hexane phase was removed and loaded onto an RP-18 column, and UV detection was conducted at 294 nm. The vitamins levels were estimated using a calibration curve with a range of 0.5–25 µg/mL (*r*^2^ = 0.9985) for vitamin A and 4–100 µg/mL (*r*^2^ = 0.9989) for vitamin E.

### 2.4. Macromolecule Modifications

#### 2.4.1. Determination of Protein Modifications

Protein modifications were quantified by the level of proteins carbonyl groups and adducts of 4-hydroxynonenal (4-HNE) with proteins.

Carbonyl group levels were estimated spectrophotometrically at 370 nm using 2,4-dinitrophenylhydrazine [43]. Concentrations are expressed in nmol/mg of protein.

Formation of 4-HNE-protein adducts was determined by ELISA [44]. Plasma samples were incubated overnight at 4 °C with monoclonal primary antibody against 4-HNE-His (anti-4-HNE-His murine monoclonal antibody, clone 4-HNE1g4). A labeled goat anti-mouse antibody (Dako, Carpinteria, CA, USA) was used as a secondary antibody. After washing, chromogen substrate was applied (3,30,5,50-tetramethylbenzidine at 0.1 mg/mL) and absorption was measured at 450 nm. Levels of 4-HNE-protein adducts were estimated using a calibration curve with a range of 1–250 pmol/mL (*r*^2^ = 0.9982). The results were normalized against total protein content and are expressed as pmol/mg protein.

#### 2.4.2. Determination of the Level of Lipid Peroxidation Products

Solid phase extraction was used for isolation of 8-iso PGF_2α_ from plasma. Quality and quantity determinations were carried out by ultra-performing liquid chromatography tandem mass spectrometry (LCMS 8060, Shimadzu, Kioto, Japan) [45]. Separation was performed on a solvent gradient from 40% to 100% acetonitrile. All samples were supplemented with an internal standard (8-iso PGF_2α_-d_4_) and analyzed in negative ion MRM mode. The transitions of precursor to the generated ion were as follows: *m*/*z* 353.2 → 193.1 for 8-iso PGF_2α_; and 357.2 → 197.1 for 8-iso PGF_2α_-d_4_. Levels of 8-iso PGF_2α_ are expressed in pg/mL.

The level of 4-hydroxynonenal (4-HNE) was determined as an O-PFB-oxime-TMS derivative using gas chromatography coupled with mass spectrometry (GC-MS/MS, Agilent Technologies, Santa Clara, CA, USA) [46]. 4-HNE was derivatized by O-(2,3,4,5,6-pentafluorobenzyl)hydroxylamine hydrochloride. Samples were deproteinized 24 h after incubation with methanol. O-PFB-oxime derivatives were extracted with hexane. The hexane layer was evaporated and N,O-bis(trimethylsilyl)trifluoroacetamide in 1% trimethylchlorosilane was added, then 1 µL aliquots were loaded onto the column. The following ions were monitored: *m*/*z* 242.0 for 4-HNE-PFB-TMS; and *m*/*z* 245.0 for 4-HNE-d_3_ derivative. Levels of 4-HNE are expressed in nmol/mL.

#### 2.4.3. Determination of the Level of Endocannabinoids

Endocannabinoid (anandamide (AEA) and 2-arachidonylglycerol (2-AG) levels were determined by LC-MS (LCMS 8060, Shimadzu, Kyoto, Japan) [32]. The solid phase extraction technique was used to obtain the analyzed compounds. Chromatographic conditions used for the separation of endocannabinoids were as follows: 1 min for initial isocratic elution with 70% acetonitrile (ACN) in water (containing 0.1% (*v*/*v*) formic acid as ionizing agent); 1–5 min linear gradient from 70% to 80% ACN; 5–15 min gradient from 80% to 88% ACN, followed by a hold at 100% ACN after 0.5 min and kept until 25 min later. Compounds were analyzed in MRM mode with the use of AEA-d_8_ and 2-AG-d_8_ as internal standards. The transitions of precursors to the generated ions were as follows: *m*/*z* 348.3 → 62.1 for AEA; *m*/*z* 379.3 → 287.2 for 2-AG; *m*/*z* 356.3 → 63.1 for AEA-d_8_; and *m*/*z* 387.0 → 295.0 for 2-AG-d_8_. Levels of endocannabinoids are expressed in pmol/mL.

#### 2.4.4. Determination of Protein Expression

Expression of proteins in PMNs (either cytosolic or membrane fractions) was determined by Western blot analysis [47]. Samples were denatured by dissolving in Laemmli buffer with 5% 2-mercaptoethanol, and boiling for 10 min. Electrophoretic separation was conducted on 10–12% gels containing SDS, then proteins were transferred onto nitrocellulose membrane, blocked with 5% skim milk, and incubated overnight with primary antibodies against either phospho-Nrf2 (pSer40), TNF-α, NF-κB (p52), (all Sigma-Aldrich, St. Louis, MO, USA), CB1, CB2, or PPARγ (all Santa Cruz Biotechnology, Santa Cruz, CA, USA) at an antibody dilution of 1:1000. β-actin was used as a loading control (antibodies against which from Sigma-Aldrich, St. Louis, MO, USA). Band visualization was carried out using the BCIP/NBT Liquid Substrate System (Sigma-Aldrich, St. Louis, MO, USA). Band intensity was quantified with a Versa Doc System and Quantity One software (Bio-Rad Laboratories Inc., CA) and normalized against respective loading controls.

### 2.5. Statistical Analysis

Data are expressed as mean ± SD and were analyzed by one-way analysis of variance (ANOVA) followed by post hoc Tukey testing using Statistica software (Statistica 13.3, Stat Soft Polska, Poland). Values of *p* ≤ 0.05 were considered significant, and only these results were discussed in detail.

## 3. Results

**Figure 1:**
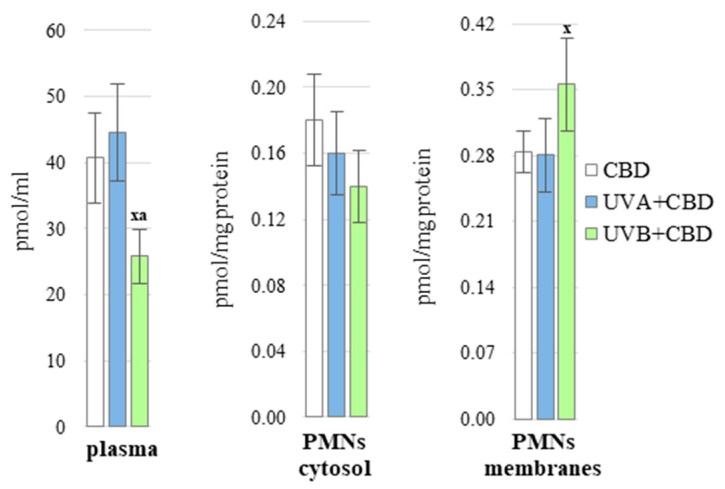
The level of CBD in blood plasma and PMNs (cytosol and membranes fraction) of nude rats in the following groups: treated with CBD (every 12 h) for 4 weeks; irradiated with UVA (every 48 h) and treated with CBD (every 12 h) for 4 weeks. irradiated with UVB (every 48 h) and treated with CBD (every 12 h) for 4 weeks. The mean values for six rats in each group ± SD are shown: x—differences vs. CBD group, *p* < 0.05; a—differences vs. UVA + CBD treated group.

Obtained results indicated that CBD penetrated the layers of the skin and was absorbed into the blood, as evidenced by its presence both in blood plasma and PMNs (Figure 1). However, after exposure to UVB radiation the level of CBD was decreased in the plasma and increased in the membrane fraction of PMNs. Thus, the presence of CBD in the blood clearly indicates the possible influence of this phytocannabinoid on metabolic processes in the body, including in the blood of rats.

**Figure 2:**
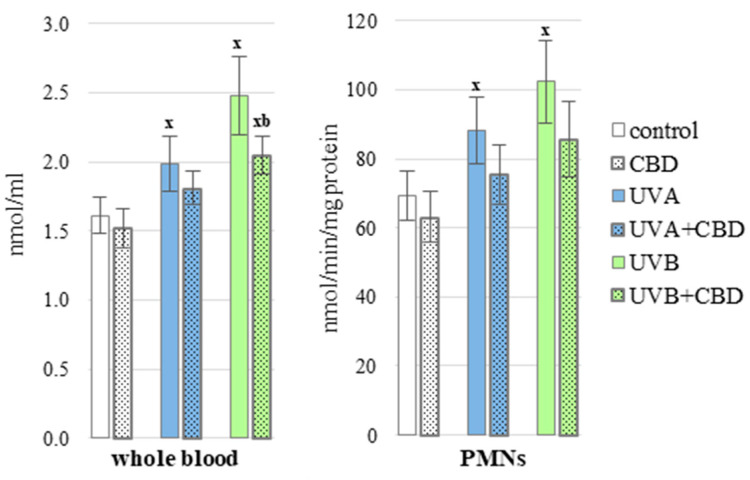
ROS levels in nude rat whole blood and PMNs in the following groups, in the following groups: control; treated with CBD (every 12 h) for 4 weeks; irradiated with UVA (every 48 h) for 4 weeks; irradiated with UVA (every 48 h) and treated with CBD (every 12 h) for 4 weeks; irradiated with UVB (every 48 h) for 4 weeks; irradiated with UVB (every 48 h) and treated with CBD (every 12 h) for 4 weeks. The mean values for six rats in each group ± SD are shown: x—differences vs. control group, *p* < 0.05; b—differences vs. UVB treated group, *p* < 0.05.

**Figure 3:**
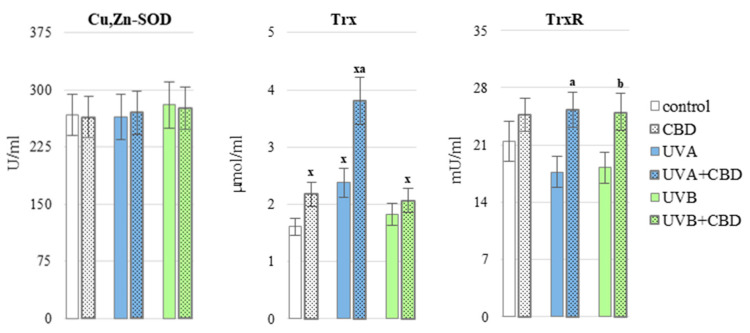
The activity of superoxide dismutase (Cu, Zn-SOD) as well as the level of thioredoxin (Trx) and the activity of thioredoxin reductase (TrxR) in the blood plasma of nude rats in the following groups: control; treated with CBD (every 12 h) for 4 weeks; irradiated with UVA (every 48 h) for 4 weeks; irradiated with UVA (every 48 h) and treated with CBD (every 12 h) for 4 weeks irradiated with UVB (every 48 h) for 4 weeks; irradiated with UVB (every 48 h) and treated with CBD (every 12 h) for 4 weeks. The mean values for six rats in each group ± SD are shown: x—differences vs. control group, *p* < 0.05; a—differences vs. UVA treated group, *p* < 0.05; b—differences vs. UVB treated group, *p* < 0.05.

**Figure 4:**
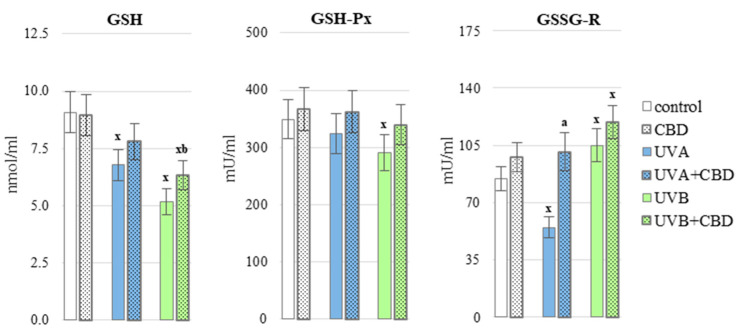
The level of glutathione (GSH) and the activity of the GSH-dependent enzymes (glutathione peroxidase (GSH-Px) and glutathione reductase (GSSG-R) in the blood plasma of nude rats, in the following groups: control; treated with CBD (every 12 h) for 4 weeks; irradiated with UVA (every 48 h) for 4 weeks; irradiated with UVA (every 48 h) and treated with CBD (every 12 h) for 4 weeks; irradiated with UVB (every 48 h) for 4 weeks; irradiated with UVB (every 48 h) and treated with CBD (every 12 h) for 4 weeks. The mean values for six rats in each group ± SD are shown: x—differences vs. control group, *p* < 0.05; a—differences vs. UVA treated group, *p* < 0.05; b—differences vs. UVB treated group, *p* < 0.05.

**Figure 5:**
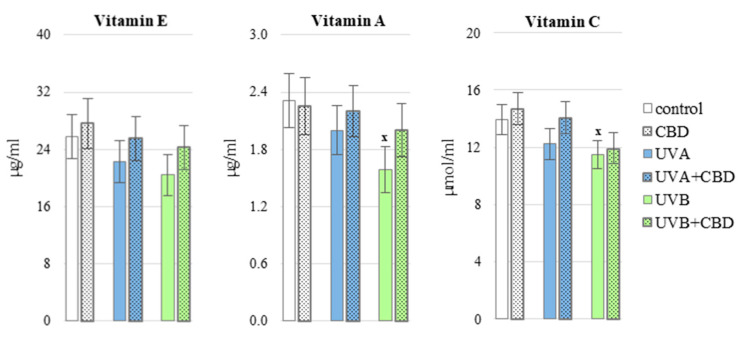
The level of vitamins E, A, C in the blood plasma of nude rats in the following groups: control; treated with CBD (every 12 h) for 4 weeks; irradiated with UVA (every 48 h) for 4 weeks; irradiated with UVA (every 48 h) and treated with CBD (every 12 h) for 4 weeks; irradiated with UVB (every 48 h) for 4 weeks; irradiated with UVB (every 48 h) and treated with CBD (every 12 h) for 4 weeks. The mean values for six rats in each group ± SD are shown: x—differences vs. control group, *p* < 0.05.

CBD partially prevented redox imbalances in the blood of rats exposed to UV radiation. This phytocannabinoid significantly decreased the ROS generation in the whole blood of animals treated with UVB compared to the group of animals irradiated only with UVB (Figure 2). Moreover CBD upregulated some analyzed antioxidants, including Trx level and glutathione reductase (GSSG-R) activity in plasma of UVA-irradiated rats as well as GSH level and thioredoxin reductase (TrxR) activity in plasma from animals exposed to UVB radiation (Figure 3 and Figure 4), it may contribute to a greater effectiveness of the glutathione and thioredoxin systems in protecting lipids against oxidative processes. The only enzymes whose activity did not change significantly under the influence of CBD was copper, zinc-dependent superoxide dismutase (Cu, Zn-SOD) and glutathione peroxidase (GSH-Px) (Figure 3). Cannabidiol applied to the skin of rats irradiated with UVA or UVB, did not cause a statistically significant increase in the level of vitamins A, E and C (Figure 5).

**Figure 6:**
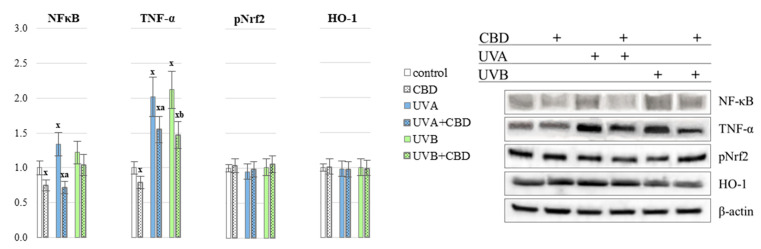
The level of NF-κB (p52) and product of its transcriptional activity—TNF-α as well as phospho-Nrf2 (p68) and product of its transcriptional activity—HO-1 in nude rats PMNs in the following groups: control; treated with CBD (every 12 h) for 4 weeks; irradiated with UVA (every 48 h) for 4 weeks; irradiated with UVA (every 48 h) and treated with CBD (every 12 h) for 4 weeks; irradiated with UVB (every 48 h) for 4 weeks; irradiated with UVB (every 48 h) and treated with CBD (every 12 h) for 4 weeks. The mean values for six rats in each group ± SD are shown: x—differences vs. control group, *p* < 0.05; a—differences vs. UVA treated group, *p* < 0.05; b—differences vs. UVB treated group, *p* < 0.05.

Changes in the redox balance in the blood of rats may be, at least in part, related to the expression and biological activity of the transcription factors Nrf2 and NF-κB assessed in PMNs, and to changes in lipid metabolism. It has been shown that UVA radiation significantly increases the expression of the transcription factor NF-κB, while the product of its transcriptional activity, the pro-inflammatory cytokine TNF-α expression was upregulated by both type of radiation. (Figure 6). In contrast, application of CBD to the skin of control and UV-irradiated rats decreased the levels of both proteins, except NF-κB expression after UVB radiation. However, neither UV radiation nor the use of CBD affected the expression of the Nrf2 transcription factor and the product of its transcriptional activity—heme oxygenase (HO-1).

**Figure 7:**
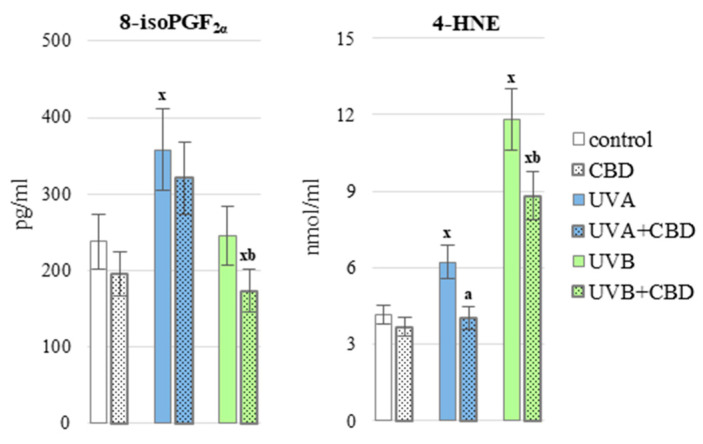
The level of 8-isoPGF_2α_ and 4-HNE in the blood plasma of nude rats in the following groups: control; treated with CBD (every 12 h) for 4 weeks; irradiated with UVA (every 48 h) for 4 weeks; irradiated with UVA (every 48 h) and treated with CBD (every 12 h) for 4 weeks; irradiated with UVB (every 48 h) for 4 weeks; irradiated with UVB (every 48 h) and treated with CBD (every 12 h) for 4 weeks. The mean values for six rats in each group ± SD are shown: x—differences vs. control group, *p* < 0.05; a—differences vs. UVA treated group, *p* < 0.05; b—differences vs. UVB treated group, *p* < 0.05.

Changes in the level and activity of redox system parameters under the influence of UV radiation indicated a shift towards oxidative processes. This hypothesis was confirmed by increased ROS-dependent lipid metabolism with an increased level of lipid peroxidation products, including 4-HNE resulting from oxidative fragmentation and 8-isoPGF2α resulting from oxidative cyclization (Figure 7). It should be emphasized that the oxidative fragmentation was much more intensified under the influence of, in particular, UVB radiation while oxidative cyclization under the influence of UVA radiation. On the other hand, the application of CBD to UV irradiated skin caused reduction in the levels of both types of lipid peroxidation products after UVB radiation and only the level of 4-HNE after UVA radiation.

**Figure 8:**
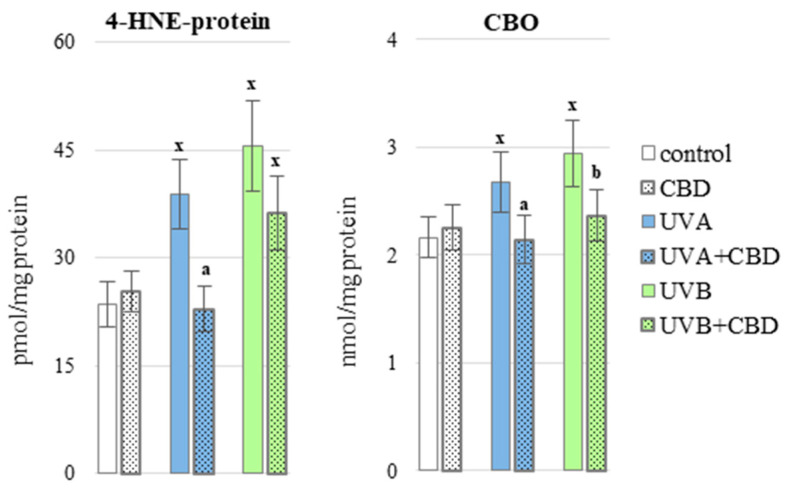
The level of 4-HNE-protein adducts and protein carbonyl groups (CBO) in the blood plasma of nude rats in the following groups: control; treated with CBD (every 12 h) for 4 weeks; irradiated with UVA (every 48 h) for 4 weeks; irradiated with UVA (every 48 h) and treated with CBD (every 12 h) for 4 weeks; irradiated with UVB (every 48 h) for 4 weeks; irradiated with UVB (every 48 h) and treated with CBD (every 12 h) for 4 weeks. The mean values for six rats in each group ± SD are shown: x—differences vs. control group, *p* < 0.05; a—differences vs. UVA treated group, *p* < 0.05; b—differences vs. UVB treated group, *p* < 0.05.

By generating oxidative stress and increasing the levels of lipid peroxidation products, UV radiation also promoted the modification of protein structure through oxidative carbonylation and the formation of protein adducts with 4-HNE, a reactive, electrophilic lipid peroxidation product. Under the influence of UVA radiation, the level of 4-HNE—protein adducts increased by about 40%, and under the influence of UVB radiation, almost twice (Figure 8). The use of CBD resulted in a significant reduction in the level of these adducts after UVA radiation. UVA/UVB radiation also increased the level of carbonylated proteins (CBO), and treatment of irradiated skin with CBD reduced the level of these modifications.

**Figure 9:**
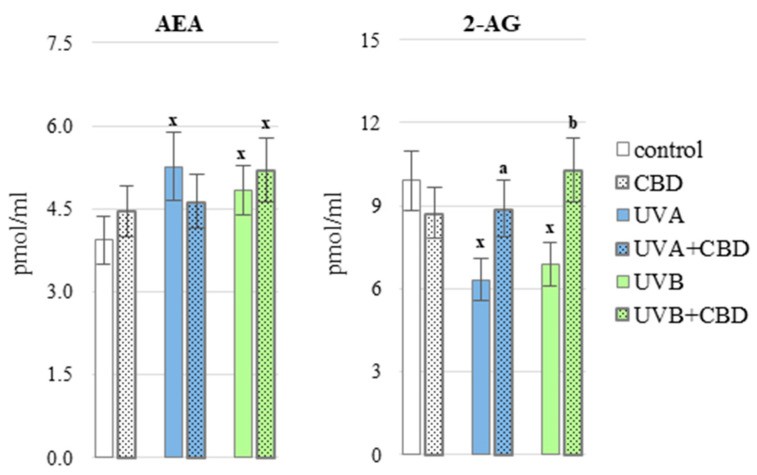
Endocannabinoids level AEA and 2-AG in the blood plasma of nude rats in the following groups: control; treated with CBD (every 12 h) for 4 weeks; irradiated with UVA (every 48 h) for 4 weeks; irradiated with UVA (every 48 h) and treated with CBD (every 12 h) for 4 weeks; irradiated with UVB (every 48 h) for 4 weeks; irradiated with UVB (every 48 h) and treated with CBD (every 12 h) for 4 weeks. The mean values for six rats in each group ± SD are shown: x—differences vs. control group, *p* < 0.05; a—differences vs. UVA treated group, *p* < 0.05; b—differences vs. UVB treated group, *p* < 0.05.

UV-induced oxidative stress also disrupted the enzymatic metabolism of lipids, as assessed by changes in the endocannabinoids level (Figure 9). UVA/UVB radiation increased the level of anandamide and decreased the level of 2-AG in the plasma of rats. In contrast, application of CBD to the skin of rats irradiated with UVA/UVB increased the level of 2-AG almost to the level in the plasma of control rats.

**Figure 10:**
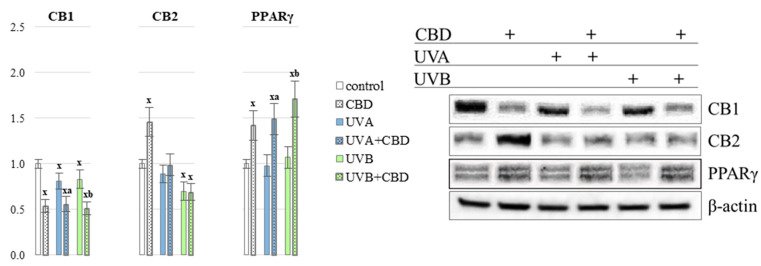
Endocannabinoids receptors expression CB1, CB2 and PPARγ in the PMNs of nude rats in the following groups: control; treated with CBD (every 12 h) for 4 weeks; irradiated with UVA (every 48 h) for 4 weeks; irradiated with UVA (every 48 h) and treated with CBD (every 12 h) for 4 weeks; irradiated with UVB (every 48 h) for 4 weeks; irradiated with UVB (every 48 h) and treated with CBD (every 12 h) for 4 weeks. The mean values for six rats in each group ± SD are shown: x—differences vs. control group, *p* < 0.05; a—differences vs. UVA treated group, *p* < 0.05; b—differences vs. UVB treated group, *p* < 0.05.

The changes in endocannabinoids level were accompanied by changes in the expression of their receptors (in PMNs) for which these compounds are agonists. The level of the CB1 cannabinoid receptor was decreased by approx. 20% under the influence of UVA/UVB radiation, while the use of CBD favored the down-regulation of the expression of this receptor by approx. 40% both in the control and after irradiation of animals with UVA/UVB. In the case of the CB2 receptor, an increase in expression by approx. 50% was observed under the influence of CBD alone in the PMNs of control rats. However, under the influence of UVB radiation and the use of UVB and CBD, a reduction of CB2 expression by about 30% was found (Figure 10). On the other hand, the expression of the PPARγ receptor was significantly up-regulated by approx. 50% in each of the groups treated with CBD (both control and UVA/B irradiation).

## 4. Discussion

UV radiation is a physical factor that, as a component of sunlight, is almost constantly present in human life, but is also used in phototherapy of skin diseases [16,48]. However, UV radiation can also pose a threat to the physiological functioning of skin cells. Therefore, extensive research is carried out on the influence of UV radiation on the metabolism of skin cells [49]. Most studies are conducted in vitro [12,50], but there are also in vivo data showing metabolic effects of long-term exposure of animal skin [51,52,53]. However, only single studies have investigated metabolic changes in internal tissues/organs [54]. It is known, that photons of UV radiation (especially UVA) penetrate deep into the dermis and have the ability to directly affect blood and lymph vessels [55]. UVB radiation, on the other hand, is responsible for many direct photochemical changes and is known to stimulate skin cells to release cytokines (and other signaling molecules) directly into the blood [56]. Moreover, both UVA radiation and UVB radiation cause overproduction of ROS in skin cells, which leads to oxidative stress resulting in oxidative modifications of DNA, proteins and lipids [12]. Due to extracellular signaling, in particular, the effects of these disturbances in cellular metabolism in the skin spread to other tissues [54,57]. They are reflected in the levels of biologically important compounds in body fluids, as evidenced by plasma proteomics and metabolomics studies, for example in the case of wound healing [58]. Therefore, it has been suggested that also the effects of UV radiation used for phototherapeutic applications may contribute to systemic metabolic changes and, consequently, affect the clinical effects of e.g., phototherapy.

The anti-inflammatory/antioxidant effects of CBD are milder than those of the commonly used corticosteroids. Therefore, CBD can be used safely in therapy supporting the treatment of skin diseases such as atopic dermatitis or psoriasis as well as skin lesions resulting from excessive exposure of the skin to sunlight or UV phototherapy.

### 4.1. UV Effect on Redox Status in Nude Rats Plasma

The results of this study confirmed that chronic exposure of rats’ skin to UV radiation, especially UVB, leads to redox imbalance in the blood as a result of increased ROS generation, especially in the whole blood and the accompanying reduction in antioxidant abilities. A similar direction of systemic redox state changes was also observed earlier in the literature [17]. Oxidizing conditions may affect the redox-sensitive transcription factor Nrf2, the expression and function of which may be impaired, as shown in previous in vitro studies in UV-irradiated skin cells [24,59]. However, these changes in expression are not supported by the results of this study on PMN fractions after UVA/UVB irradiation of the animal skin, but it is believed that not only changes in the biological activity of Nrf2 are important. Maintaining an appropriate metabolic interaction between Nrf2 and NF-κB after irradiation with UV rays also influences metabolic homeostasis [8,60]. It is known that the lack of Nrf2 response in PMNs observed in this study may potentiate the activity of NF-κB, leading to increased production of pro-inflammatory cytokines including TNF-α, which is significantly increased in PMNs of UV-irradiated rats, usually resulting in cellular receptors activation and further initiation of intracellular phosphorylation events that coordinate cell signaling [61]. In response to acute inflammation following UV irradiation, NF-κB also stimulates the mitochondrial expression of NADPH oxidase, as observed in whole blood and PMNs fraction of UV-irradiated rats, which is the main source of endogenous ROS [62].

The above situation promotes the enhancement of the effect of ROS on both small molecules and macromolecules such as lipids as well as peptides and proteins, what could explain the increased carbonylation of protein molecules and increased levels of lipid peroxidation products, observed in this study. As a consequence, the nucleophilic elements of the protein structure are additionally modified by the electrophilic element of 4-HNE with the formation of 4-HNE-protein adducts. Thus, under oxidation conditions induced by UV radiation, proteins including antioxidant enzymes, modified by ROS and 4-HNE have a lower biological efficiency [63], which is manifested by a decrease in GSH-Px activity after UVB radiation. The accompanying reduction in the level of GSH favors the down-regulation of antioxidant effectiveness toward lipids and the intensification of pro-oxidative effects. Especially the decrease in GSH level intensifies the reduction of other non-enzymatic antioxidants (especially vitamins A and C), with which GSH cooperate in maintaining redox balance and preventing lipid modifications [64], observed as enhanced level of 4-HNE. In contrast, previous studies in mice have shown that a single exposure to UVA radiation causes a significant reduction in the activity of glutathione reductase and catalase and a reduction in the level of GSH in the skin, blood and liver, while exposure to UVB radiation contributes to a reduction in the activity of superoxide dismutase, glutathione peroxidase and GSH level [65]. It is believed that this is related to the depletion of antioxidant resources and an increase in the level of lipid peroxidation products, especially in the liver [57]. Literature data also indicate that skin cells exposed to UV radiation produce both signaling molecules and stress proteins, which may also affect other tissues [66,67]. Despite the direct changes in the redox balance in the internal tissues caused by the irradiation of the skin, it is believed that there is a possibility of penetration into the blood of directly damaged biomolecules, as well as de novo generated signaling molecules. In addition, post-translational modifications of proteins caused by the action of UV radiation on skin cells may enter the bloodstream and thus affect metabolic changes in the blood and internal organs [67].

The oxidative stress stimulated by UV radiation leads to the changes in lipid metabolism, resulting not only from the overproduction of ROS, but also the increased activity of a number of enzymes metabolizing lipids with the generation of their derivatives including endocannabinoids and eicosanoids [68,69]. The consequence are changes in endocannabinoid levels, such as anandamide increase and a decrease in 2-AG levels after UVA/UVB irradiation, leading to down-regulation of CB1 receptor after UVA/UVB irradiation, and CB2 receptor after UVB irradiation in PMNs. Given that cannabinoid receptors are responsive for regulation of ROS and TNF-α levels [70], such situation may additionally modify redox balance and inflammation. Due to the fact that UV radiation particularly impacts upon antioxidants involved in lipid protection, it has even been suggested that by modifying lipid metabolism, UV radiation may change the structure of cell membranes and blood vessel walls [54].

### 4.2. CBD Protection against UV-Induced Disturbances

In order to protect skin cells against the effects of metabolic disorders, various protective compounds have been proposed, including polyphenols, rutin, ascorbic acid and phytocannabinoids (CBD and PEA) [21,50,71,72]. Especially taking into account the anti-inflammatory/antioxidant effects of CBD, which, however, are milder than that of e.g., corticosteroids, CBD, like another phytocannabinoid—PEA [73], can be used in therapy supporting both local skin problems, e.g., caused by the development of diseases such as atopic dermatitis or psoriasis, as well as resulting from solar radiation or phototherapy with the use of UV radiation. In the context of metabolic changes in the blood caused by irradiation of the skin with UV rays, it was decided to determine whether cannabidiol, an anti-inflammatory and antioxidant phytocannabinoid protecting skin cells in vitro and ex vivo [5,23], can also prevent the transmission of metabolic changes in the blood of hairless rats.

It is known that the percutaneous application of the preparation ensures a constant level of the compound used in the body and a high degree of sensitivity to the compound used [30]. Since CBD is a lipophilic molecule [74] it has previously been shown that it tends to accumulate only in the stratum corneum and penetrates poorly into deeper layers [75]. Therefore, the ability of CBD to enter the bloodstream was verified by assessing its level in the circulation. This is all the more important as CBD has been tested for several years in supporting phototherapy in the treatment of psoriasis, which despite the fact that it is a skin disease, but the resulting metabolic disorders affect the entire body [5,76].

The results of this study confirm that CBD when applied chronically to rat skin penetrates the bloodstream and has been shown to be present in blood plasma and blood cells such as PMNs. However, the application of CBD to the skin irradiated with UVB causes the plasma levels of CBD to be lower, and the membrane fraction of PMNs to be higher than the corresponding levels measured in the group of rats treated only with CBD. This discrepancy may result from the lipophilic nature of CBD and, consequently, from the tendency to accumulate in cell membranes, which was previously observed after in vitro UVB irradiation of keratinocytes [21]. As a result, slightly less CBD reaches the bloodstream than would be expected from the amount applied to the skin, favoring lower plasma levels of CBD.

Consequently, the effectiveness of the antioxidant effect of CBD at the blood level is observed, which leads to the reduced levels of ROS in whole blood of rats exposed to UVB. It is known that CBD, like other antioxidants, breaks the chain reactions of free radicals, trapping them or transforming them into less active forms and reducing the activity of ROS-producing enzymes [19]. Such changes were also found earlier after applying CBD to UV irradiated keratinocytes both from psoriatic patients and healthy people [5], and in a model of renal nephropathy in mice treated with cisplatin [77]. The reduction of ROS levels by CBD may also result from the direct interaction of this phytocannabinoid with membrane receptors, including cannabinoid receptors, of which cannabidiol is a weak agonist also in rats [78,79]. Moreover, it is known that CBD can also activate, depending on the concentration, other G-protein coupled receptors, especially TRPVs and PPARs [78,80,81]. In addition to direct receptor activation, CBD also acts indirectly by enhancing the biosynthesis of 2-AG, an endocannabinoid agonist of cannabinoid receptors [82]. As a consequence, a decrease activation of CB1 receptors is observed in PMNs, which may indicate down-regulation of ROS and TNF-α generation [70]. However, upregulation of the CB2 receptor by CBD in control PMNs also indicates downregulation of ROS production and confirms an antioxidant shift in the redox state of cells. Thus, the results of this study indicate that CBD, by modulating the activation of cannabinoid receptors, may contribute to the antioxidant response in the blood.

Taking into account the influence of topical CBD application on blood cell receptors, it can be assumed that also other phytocannabinoids present as dietary components in consumed food plants [83] penetrating into the blood, they can affect, among others on G-protein coupled receptors [83,84] and therefore through cannabinomimetic effects on the body’s metabolism.

Moreover, both CBD and 2-AG activate the nuclear PPARγ receptor, thus contributing to anti-inflammatory and antioxidant effects, and may also be involved in the induction of transcription factors such as NF-κB and Nrf2 [24,85,86], which play a key role in tissue protection and repair, including the activation of Nrf2 and/or inhibition of NF-kB [87,88]. In addition, CBD induced the expression of several Nrf2 target genes, with heme oxygenase 1 (HO-1) being the gene and the protein most upregulated by CBD [23]. CBD, acting as a transcriptional repressor, controls cell proliferation and differentiation through DNA methylation, an important element in epigenetic regulation, with low concentrations of CBD promoting cell survival, while high concentrations increasing the likelihood of cell death [89,90].

CBD is known to interact with the Rel homology domain of the p65 subunit of NF-κB, which ultimately leads to its degradation [91,92]. The occurrence of such a situation may explain the problem with the determination of the p65 subunit level in the PMNs of rats treated with CBD, which was observed in the present study. However, it is known that proteasomal degradation of NF-κB (p65) can inhibit the expression of pro-inflammatory genes, cyclooxygenase (COX2), and some other pro-inflammatory mediators including TNF-α, and its degradation can also inhibit pro-inflammatory signaling mediated by NF-κB [93]. It has been suggested that participation in this pathway may also be related to impaired translocation of NF-κB subunits to the nucleus, as well as to degradation/phosphorylation of IκBα catalyzed by ERK1/2 and p38 MAPK [94,95]. Moreover, CBD was found to inhibit the EGF/EGFR pathway in cancer cells [96], which also inhibits the activation of the pro-inflammatory NF-κB pathway. CBD may also influence the activity of the NF-κB by inhibiting the UV-induced expression of heat shock proteins [66]. On the other hand, PPARγ cooperating with Nrf2, controls the expression of genes encoding antioxidant proteins, including superoxide dismutase and HO-1 [97]. However, no changes in the expression of Nrf2 are observed in this study as well the changes in the level of HO-1 and the activity of Cu, Zn-SOD.

The antioxidant effect of CBD is also evident in the partial prevention of peroxidation of PUFAs present in lipids, which can lead to oxidative fragmentation to form predominantly α,β-unsaturated aldehydes, including 4-HNE, as well as oxidative cyclization to prostaglandin derivatives, including the most frequently rated 8-isoPGF2α [98,99]. The formation of these lipid peroxidation products may have a direct impact on the composition and structure of cell membranes, and thus on their ability to perform biological functions [100]. Therefore, by lowering the levels of reactive aldehydes and prostaglandin derivatives generated by exposure to UV radiation, CBD reduces the effects of oxidative stress. In addition, CBD by lowering the level of 4-HNE protects peptides and antioxidant proteins (e.g., GSH, Trx, TrxR and GSH-Px), which, due to active nucleophilic elements, form adducts with 4-HNE, which reduce their biological activity [101].

Taking into account the results of this study and the observed synergism of the action of plant metabolites, it is possible to suggest a way to intensify the biological activity of cannabidiol by using it, for example, with flavonoids or whole plant extracts containing both types of compounds. This can enhance the interaction of CBD with receptors, and as low molecular weight compounds, flavonoids can facilitate the crossing of the skin/blood brain barrier and absorption after oral administration, so the effectiveness of CBD may increase regardless of the route of administration [102]. However, considering the possibility of such an approach, it should be remembered that, for example, isoflavones, by binding to estrogen receptors, affect the production of sex hormones, which may be a problem with the chronic use of such combinations.

## 5. Conclusions

In the context of the effects of CBD demonstrated in this study, it can be concluded that due to the penetration of this phytocannabinoid from the site of application (skin) into the blood, CBD has the ability to partially alleviate the systemic oxidative stress caused by UV radiation from the skin of nude rats as observed in the blood of animals. The protective effect of CBD has a positive effect on the structure and function of lipids and proteins. This is reflected in the levels/activities of the redox components as well as in the reduced lipid metabolism dependent on both ROS and enzymes. Thus, the results of this study indicate that CBD can prevent metabolic disorders induced by UV radiation not only at the skin level, as previously shown [22], but also in the body as a whole. 

Results of this study together with the fact that UV radiation contributes, among others, to the development of skin cancer, including melanoma, and the study on mice with induced melanoma, showing that intraperitoneal administration of CBD reduces tumor growth and prolongs the life of animals [103], suggests the possibility of using CBD both for therapeutic purposes (counteracting the effects of UV radiation) and supporting therapies related to skin diseases, including cancer.

## Figures and Tables

**Table 1:** Biochemical parameters in plasma of rats which skin was irradiated with UV and treated with CBD.

Parameters	Control	CBD	UVA	UVA + CBD	UVB	UVB + CBD
total protein [g/dL]	5.41 ± 0.44	5.40 ± 0.48	5.25 ± 0.55	5.35 ± 0.56	5.40 ± 0.58	5.35 ± 0.53
Na^+^ [mmol/L]	85.1 ± 3.1	85.3 ± 3.2	86.9 ± 3.4	85.2 ± 3.2	86.9 ± 3.5	86.6 ± 3.2
K^+^ [mmol/L]	4.52 ± 0.22	4.51 ± 0.21	4.54 ± 0.24	4.45 ± 0.23	4.62 ± 0.26	4.54 ± 0.23
glucose [mg/dL]	220 ± 12	224 ± 13	214 ± 12	224 ± 13	227 ± 13	226 ± 13
AST [U/L]	81 ± 4	78 ± 5	86 ± 5	82 ± 4	79 ± 5	80 ± 5
ALT [U/L]	43 ± 2	41 ± 3	46 ± 3	45 ± 3	46 ± 4	43 ± 3
triglycerides [mg/dL]	85 ± 6	85 ± 7	88 ± 7	87 ± 8	93 ± 8	87 ± 7
cholesterol [mg/dL]	74 ± 5	74 ± 6	73 ± 6	73 ± 5	75 ± 7	73 ± 5
urea [mg/dL]	43 ± 3	41 ± 4	46 ± 4	46 ± 4	45 ± 4	43 ± 3

## Data Availability

The data presented in this study are contained within the article.
